# Translational opportunities in animal and human models to study alcohol use disorder

**DOI:** 10.1038/s41398-021-01615-0

**Published:** 2021-09-29

**Authors:** Steven J. Nieto, Erica N. Grodin, Claudia G. Aguirre, Alicia Izquierdo, Lara A. Ray

**Affiliations:** 1 Department of Psychology, University of California at Los Angeles, Los Angeles, CA USA; 2 Department of Psychiatry and Biobehavioral Sciences, University of California at Los Angeles, Los Angeles, CA USA

**Keywords:** Scientific community, Addiction

## Abstract

Animal and human laboratory paradigms offer invaluable approaches to study the complex etiologies and mechanisms of alcohol use disorder (AUD). We contend that human laboratory models provide a “bridge” between preclinical and clinical studies of AUD by allowing for well-controlled experimental manipulations in humans with AUD. As such, examining the consilience between experimental models in animals and humans in the laboratory provides unique opportunities to refine the translational utility of such models. The overall goal of the present review is to provide a systematic description and contrast of commonly used animal paradigms for the study of AUD, as well as their human laboratory analogs if applicable. While there is a wide breadth of animal species in AUD research, the paradigms discussed in this review rely predominately on rodent research. The overarching goal of this effort is to provide critical analysis of these animal models and to link them to human laboratory models of AUD. By systematically contrasting preclinical and controlled human laboratory models, we seek to identify opportunities to enhance their translational value through forward and reverse translation. We provide future directions to reconcile differences between animal and human work and to improve translational research for AUD.

## Introduction

Alcohol use disorder (AUD) is highly prevalent in the United States [1] and incurs substantial individual and societal costs [2]. AUD is considered a heterogenous disorder involving complex etiologies and mechanisms. Animal and human experimental models play an integral role in understanding these multidimensional aspects of AUD. However, there is growing concern as to the degree of confidence we can have in animal experiments and their behavioral endpoints in terms of their translational utility [3]. Similarly, human laboratory studies have been criticized for their lack of predictive utility of clinical trials outcomes [4]. The absence of predictive validity of some animal models has contributed to a decline in psychiatric drug development programs [5].

Compared to human laboratory models, experimental studies in animals allow for precise experimental control over several parameters, including genetics, the influence of environment (upbringing, stress), and previous experience with alcohol, drugs, and rewards in general. The use of experimental animals in research allows for the examination of neurochemical, neurobiological, and neurophysiological factors that may ultimately translate to disease states in humans. These correlates can then facilitate the development of novel therapeutic targets for complex, heterogeneous disorders such as AUD. However, animal models of AUD are not without criticism. Notably, genetically diverse rat strains (outbred) do not readily consume alcohol and reach minimal blood alcohol concentrations. While several experimental manipulations were developed to induce alcohol drinking or self-administration behaviors that result in pharmacologically meaningful ethanol intake, these manipulations introduce a host of other influential factors (e.g., stress, palatability) that may limit generalizability. Rodents genetically bred to consume high levels of alcohol [6] have also been scrutinized for their lack of generalizability.

While it is important to acknowledge that no animal model of addiction fully encapsulates the human condition, experimental work in animals permit the investigation of specific and observable elements of the addiction process. Thus, animal models are most likely to have construct or predictive validity when the model mimics the specific signs or symptoms associated with a given disorder. The focus of animal models should not be to fully emulate the whole syndrome but rather to inform on specific domains that can be validated across species. That is, the goal of the animal model is to achieve a better understanding of the biological dysfunction that contributes to the disorder and to ensure that this level of understanding can be translated to novel treatments. Equally important, animal models should be validated beyond mirroring behaviors. Demonstrating shared circuit abnormalities is critical to establish face validity. In several areas of neuroscience, including addiction, there is tremendous evidence for conservation across species for basic behaviors and underlying circuit-, cellular-, and molecular-based mechanisms.

Human laboratory studies have also been developed to study discrete aspects of AUD phenomenology. Herein, we argue that human laboratory models provide a “bridge” between preclinical and clinical studies of AUD by allowing for well-controlled experimental manipulations in humans with AUD. As such, examining the consilience between experimental models in animals and humans in the laboratory provides unique opportunities to refine the translational utility of such models. This would allow alcohol researchers to more effectively leverage preclinical findings to clinical applications and vice versa. For example, laboratory-controlled alcohol administration permit investigations into the pharmacokinetic and pharmacodynamic responses to alcohol, which are proposed AUD risk factors. Human self-administration permits objective behavioral assessment of alcohol consumption, motivation, and compulsive use which are relevant to addiction phenomenology. Given that progression to addiction is accompanied by increasing salience of drug-paired cues, cue-reactivity assessments have also been implemented into human laboratory models. Importantly, such models can then be leveraged to treatment development and serve as early efficacy markers for promising treatments, both behavioral and pharmacological [4, 7].

In the present review, we provide a systematic description and contrast of commonly used animal paradigms for the study of AUD, as well as their human laboratory analogs. Previous reviews from our group have emphasized the opportunities to improve translational science for AUD [7–9]. However, this is a unique endeavor in that we focus on preclinical models in detail and discuss each model from the perspective of clinical and translational research. While there is a wide breadth of animal species in AUD research, the paradigms discussed in this review rely predominately on rodent research. The overarching goal of this effort is to provide critical analysis of these animal models and to link them to human laboratory models of AUD. The premise is that addiction phenomenology is inherently a human process and that capturing key phenomenological aspects of addiction under controlled conditions, in humans and rodents, poses unique challenges. By systematically contrasting preclinical and controlled human laboratory models, we seek to identify opportunities to enhance their translational value through forward and reverse translation (see Table 1). We provide future directions to reconcile differences between animal and human work to improve translational research for AUD.

**Table 1:** Comparison on animal and human models applied to alcohol use disorder.

Paradigm	Animal Model	Human Model	Opportunities for Translation
CONDITIONED PLACE PREFERENCE (CPP)	X	X	Limited utility in human models
NONCONTINGENT ALCOHOL ADMINISTRATION	X	X	Lack of consilience between outcomes (e.g., subjective responses in humans versus consumption/alcohol choice in rodents)
ACQUISITION OF OPERANT SELF-ADMINISTRATION	X	--	Parallel human model is needed
PROGRESSIVE RATIO SELF-ADMINISTRATION	X	X	Recently developed human models yet to be leveraged
CUE-INDUCED REINSTATEMENT	X	X	Further understanding on the degree of variability in the extent of cue-reactivity in human models is needed
DRUG-PRIMED REINSTATEMENT	X	X	Relevance to treatment context is unclear
STRESS-INDUCED REINSTATEMENT	X	X	Dysfunction in negative emotionality domain may predict relapse
VOLUNTARY ABSTINENCE RESINSTATEMENT	X	X	Utility in animal models remains to be seen
REVERSAL LEARNING	X	X	Experimental studies in human models are critically lacking
BEHAVIORAL ECONOMICS	X	X	Unclear whether behavioral economic indices predict clinical outcomes / treatment response

### Experimental paradigms used to model alcohol reward and intake

#### Conditioned place preference

**Fig. 1:**
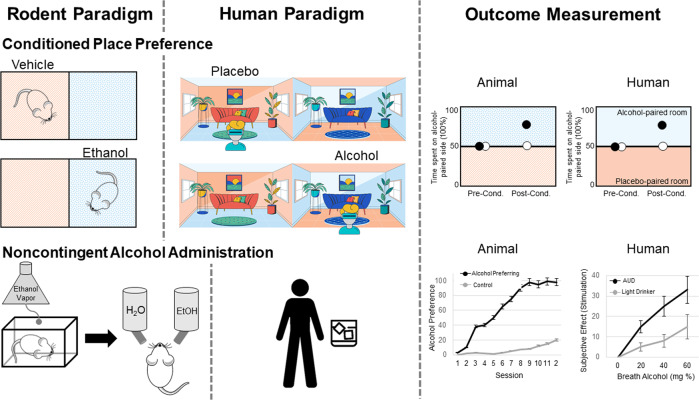
Depicts rodent and human paradigms and outcome measurement for conditioned place preference and noncontingent alcohol administration. In the rodent conditioned place preference paradigm, ethanol is paired with one chamber, while vehicle/saline is paired with the other chamber. On testing, time spent in each chamber is used to measure the rewarding value of alcohol. In the human conditioned place preference paradigm, virtual reality (or real-world settings) is used to pair a room with alcohol or placebo. On testing, time spent in each chamber is used to measure the rewarding value of alcohol. One common paradigm for noncontingent alcohol administration in rodents is the ethanol vapor chamber, used to induce ethanol dependence. Rodents are then tested for alcohol preference using the two-bottle choice test, where they can choose between ethanol or water solutions. In humans, alcohol administration is often focused on subjective responses to alcohol during ascending and descending blood alcohol levels.

Conditioned place preference (CPP) is a form of associative (Pavlovian) learning used to measure the motivational effects of stimuli/cues, or contexts. In this approach, the rewarding value of alcohol is measured by the degree to which an organism spends time in an environment (place preference) or prefer a flavored solution (taste preference) that has been paired with alcohol (see Fig. 1). It has been proposed that the behavioral loss of control over alcohol drinking that occurs in humans may be a consequence of the attraction to conditioned alcohol-paired stimuli via learning processes involved in CPP. CPP experiments are one of the most frequently used paradigms in clinical trials to test the abuse potential of new drugs. However, CPP does not directly probe how much an animal is willing to pursue the reward (i.e. operant, goal-directed behavior). Relative to operant self-administration, CPP in rodents requires passive drug administration, which results in distinct neurobiological effects relative to drug delivery that is not controlled by the animal.

CPP experiments in animals have primarily been conducted using mice and rats, although some studies show CPP in rhesus macaques and zebrafish. These procedures rely on the conditioning of an environmental or sensory context with the hedonic effects of alcohol, and the goal is to measure the extent to which the animal prefers or avoids the alcohol-associated environment or tastant while not under the influence of alcohol (see [10] for an in-depth review of CPP protocols and studies). Establishing alcohol CPP is sensitive to the number of conditioning sessions, with repeated administration resulting in preference [11] compared to a single alcohol pairing session [12].

The CPP paradigm has been translated to human studies [13–16]. Compared to the preclinical models, establishing CPP in humans requires a considerable amount of space and time commitment. One approach to make CPP studies more convenient in humans has been to use virtual reality to produce the environment and reward pairings (see Fig. 1) [13]. In healthy individuals, food, money, and arbitrary point rewards have all been demonstrated as effective rewards to induce a conditioned place preference [14, 16]. In healthy controls, intolerance to uncertainty, a personality trait which reflects the preference for familiar over uncertain choices that has been implicated as a vulnerability factor for addictive disorders, predicts “reward chasing” in the virtual CPP paradigm [17]. The conditioned place preference paradigm has also been used in human social drinkers [15]. Non-dependent individuals were able to develop a behavioral preference for a room paired with alcohol administration; however, this preference was only developed after multiple pairing sessions, which differs from findings in animal models. Recent human data indicates that contexts that are associated with heavy drinking induce craving, over and above other factors, such as negative affect [18]. However, from a human laboratory perspective, it is important consider feasibility of the model and strength of the alcohol main effect. Further, how proximal is CPP to the core phenomenology of addiction. Taking these factors into consideration, it may be that the CPP approach is limited by its time-intensive nature and the modest alcohol main effect. The ultimate application of the model hinges on its centrality to addiction theory and possibly to underlying biomarkers. To that end, the CPP approach is rather limited in its current format in humans (see Table 1).

The CPP paradigm can be leveraged to enhance our understanding of the heterogeneity in AUD. Preclinical models can utilize CPP in combination with dependence induction techniques to phenotype animals based on place preference behaviors. The rationale for this approach is that excessive alcohol intake increases incentive salience to alcohol-related cues, thus enhancing motivation for further alcohol consumption. Phenotyping animals and humans based on behaviors that capture incentive salience is a priority for research (i.e. Alcohol Addiction Research Domain Criteria) and clinical (Addictions Neuroclinical Assessment) frameworks to understand heterogeneity in AUD. Another future direction that would improve the construct validity of the paradigm involves examining the relationship between CPP behaviors to alcohol-seeking behaviors (i.e., oral alcohol administration / self-administration).

#### Noncontingent alcohol administration

The two-bottle choice preference paradigm is used often to assess the rewarding properties of alcohol [19, 20]. It is a noninvasive, non-operant self-administration method during which the animal is given the choice to voluntarily consume ethanol versus a non-ethanol beverage (usually water) orally, with either open or limited access (see Fig. 1) [21]. The two-bottle choice procedure is also sometimes used to mimic the alcohol deprivation effect as it is commonly administered intermittently. The most common version of the two-bottle choice procedure is a chronic intermittent access schedule, in which rodents have 24-hour ethanol access, 3 days per week, followed by repeated periods of deprivation (usually every other day). The animal’s preference for alcohol is represented by the percentage of total daily fluid consumed from the alcohol bottle. There is a substantial evidence demonstrating that intermittent access to ethanol gradually enhances intake in rodents across a variety of methods [22–24], which mimics the transition from moderate, social drinking, to the excessive consumption of alcohol, often seen in heavy drinkers. Moreover, the intermittent access procedure has also been used as a model of binge-like drinking in rodents, as roughly one-third of total ethanol consumed in a session occurs during the first 30 minutes, resulting in blood ethanol concentrations over 80 mg% [24].

Several criticisms regarding this model have been raised, particularly with regard to its ability to capture binge-drinking or dependence-driven ethanol intake typically seen in humans with AUD [22, 25]. Specifically, this model does not result in behavioral effects following long-term withdrawal nor does it typically produce alcohol deprivation effects, indicating that the intermittent access procedure may better model heavy/problematic drinking or mild AUD rather than moderate or severe AUD. As a result, several variants of this model have been introduced, including ethanol vapor exposure and passive intragastric infusion, in an effort to engender dependence on alcohol and excessive levels of voluntary ethanol intake meant to emulate problematic drinking patterns seen in humans (i.e., increase external validity). Despite the criticisms of this model, it has shown three types of validity: (1) face validity: given the similarity in drinking patterns seen in humans with mild AUD [26, 27]; (2) construct validity: given the high correlation of ethanol intake levels, BECs, and neuroplastic effects [28, 29]; and (3) predictive validity: given that drugs used for the treatment of alcoholism such as, naltrexone and acamprosate, suppress alcohol intake in this model [28, 29]. These findings show the two-bottle choice procedure is a reliable and efficient method of alcohol administration in animals by promoting voluntarily ethanol consumption that may yield clinically relevant ethanol consumption patterns and dependence.

On the side of human experimentation, oral alcohol administration is a well-established paradigm. Early studies used predefined dosing methods based on body weight and sex, which resulted in wide variability in blood alcohol concentrations [30]. More recent methods use mathematical models for more precise and standardized alcohol dosing regimens [31, 32]. The primary advantages of oral alcohol challenges are that they are easily implemented and ecologically valid. The target intoxicating dose is typically 0.08 g/dl; however, variability in blood alcohol concentrations, particularly time to peak BAC, is a notable disadvantage that adds methodological noise. Intravenous alcohol administration methods were developed to further enhance precision over BAC. In these experiments, a 5–6% alcohol solution is delivered intravenously at an infusion rate that considers participants’ weight and sex. A physiologically-based pharmacokinetic model more accurately estimates BAC based on participant characteristics like sex, height, weight, and age [33]. Model parameters can self-correct based on observed BAC values. The Computerized Alcohol Infusion System (CAIS) implements this model allowing fine-grained control over the ascending BAC limb trajectories and, to a lesser extent, descending limb trajectories [34, 35]. Furthermore, BAC can be maintained at a steady state over an extended period using alcohol clamp procedures [36, 37]. This paradigm allows for robust assessment of acute tolerance or sensitization to alcohol’s effects, in addition to behavioral tasks while at a stable BAC. At the expense of ecological validity, intravenous administration allows for greater experimental control and the eliminations of alcohol-related sensory cues. Relative to oral alcohol administration, intravenous challenges are significantly more expensive largely due to medical costs [9].

While the overall goals of the two-bottle choice procedure are for rodents to consume alcohol at pharmacologically relevant levels and/or to measure alcohol preference over another reward, alcohol administration in humans is more focused on subjective responses to alcohol during ascending and descending blood alcohol levels (see Fig. 1). In humans, subjective response to alcohol has emerged as a candidate endophenotype for AUD [38]. That is, differences in subjective response to alcohol can predict AUD risk. For obvious reasons, subjective responses to alcohol are difficult to assess in rodents. Nonetheless, outcome variables in human alcohol administration studies encompass subjective experiences to the sedative and stimulant effects of alcohol. Animal models to measure sensitivity to alcohol can be used as a parallel to model these subjective responses to alcohol in human. For example, sensitivity to the rewarding effects of alcohol can be measured using a combination of two-bottle choice procedures and place and taste conditioning paradigms. Sensitivity to alcohol’s sedative effects is largely captured by assays that resemble ataxia and balance in rodents, namely the rotarod task and loss of righting reflex.

The integration of animal and human alcohol administration models clearly suggests limitations to both. In simple terms, dosing and endpoints are widely different across the two species/methods. On the human side, subjective response to alcohol, while predictive of long-term alcohol use, may be less proximal to AUD than self-administration models [39, 40]. For AUD, we argue that the gold-standard endpoint should be alcohol consumption under a host of conditions (e.g., progressive ratio self-administration, choice behavior between alcohol consumption and social reward/monetary reward; consumption under conditions of stress and cue presentation, etc.). To that end, focusing on more proximal determinants of AUD, both in human and animal models may ultimately increase the ‘signal-to-noise ratio’ and advance translational science of AUD (see Table 1).

### Experimental paradigms to model alcohol reinforcement

#### Operant self-administration

**Fig. 2:**
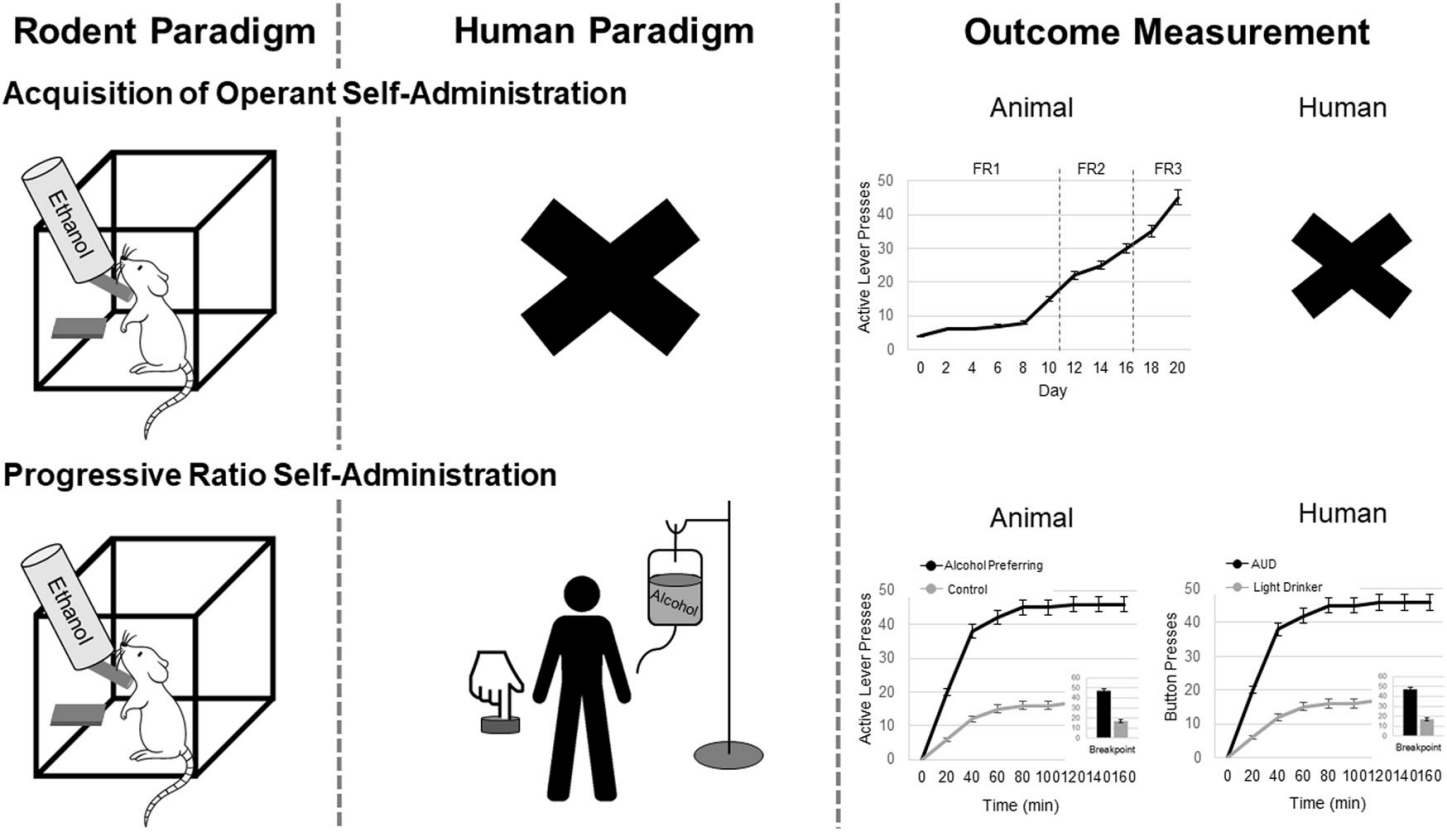
Depicts rodent and human paradigms and outcome measurement for the acquisition of operant self-administration and progressive ratio self-administration. Rodents acquire operant self-administration through the pairing of an active level press with access to an ethanol solution. Over time, rodents learn to acquire this self-administration behavior. Currently, there is no human paradigm which parallels the acquisition of self-administration. In the rodent and human progressive self-administration, the response requirement to obtain an alcohol delivery gradually increases during the operant session. The maximum number of responses the rodent/human makes in order to receive alcohol is referred to as the break point. This type of schedule reflects an animal’s motivation to obtain an alcohol delivery.

Alcohol self-administration is a procedure in which an animal or human subject performs a response, such as pressing a lever or button, which delivers a dose of alcohol. The route of alcohol delivery varies; however, oral delivery is most common in alcohol self-administration (see Fig. 2). Operant self-administration behaviors are also sensitive to environmental and pharmacological manipulations. The most basic assumption of this approach is that alcohol functions as a reinforcer, that is, it increases the likelihood of the behavior that produces it. Thus, alcohol self-administration is viewed as an operant response reinforced by the effects of alcohol. Operant behaviors are sensitive to the nature of the relationship between response and reinforcer. This relationship is known as the schedule of reinforcement and describes requirements such as how many responses are required to produce a reinforcer, how much time must pass before the next reinforcer becomes available, and what cues, if any, signal the availability of reinforcement. Several schedules of reinforcement that were originally developed for food reinforcement have been adapted to the study of alcohol [41, 42].

Schedule-induced polydipsia is a commonly used method to initiate operant self-administration, which uses scheduled delivery of food and alcohol reinforcement, resulting in excessive drinking [43]. Typically, animals are food restricted to 85% of their free-feeding body weight and placed in operant chambers, requiring the animal to lever press for a food reward delivered using a variety of interval schedules (e.g. fixed interval, variable interval, fixed time), followed immediately by fluid delivery. However, more recent applications of this method of administration no longer require lever pressing for food and fluid delivery, but have pellets delivered automatically based on a fixed time interval. Alcohol concentrations are commonly increased in a stepwise manner (e.g. 0.5 g/kg, 1.0 g/kg, 1.5 g/kg) for 30 days, once a maximum concentration is reached the induction period is completed and the animals are placed under “open access” in which they have water to ethanol and water for 22 h./day with pellets still available in a fixed ratio 1 schedule [44, 45]. Due to the many parameters (i.e. time of day, duration of access, feeding conditions, non-flavored vs. flavored alcohol, free alcohol access vs. operant responding) that can be manipulated in this model, several variations have been implemented in an attempt to increase daily intake, such as, using multiple discrete self-administration sessions during the “open access” period, increasing the ethanol concentration at a much faster rate (i.e. every 3–7 days), and flavoring the alcohol solution [46, 47].

There are several concerns when using this model for alcohol induction including, the lack of specificity, since polydipsia occurs when other liquids, not just alcohol, are made available. Animals are also typically food restricted, although some studies have removed this requirement, which makes unclear what the motivation to drink is, since it could be attributed to the pharmacological effect of alcohol or its caloric value. Moreover, operant self-administration requires a long-training period and some animals do not develop stable response patterns despite extensive training. Despite these criticisms, this model can result in persistent high alcohol intake if the experimental parameters are optimized to facilitate the transition from positive to negative reinforcing properties as alcohol (e.g. stress relief), which is thought to underlie alcohol addiction, even after the reinforcement schedule is removed [43]. Operant self-administration also offers flexibility for behavioral assessment, including procedures for measuring motivation for alcohol and continued use of alcohol despite aversive consequences. Importantly in terms of translational work, individual differences in average alcohol intake in non-human primates result in light, moderate, and heavy drinkers, with only a portion developing chronically high alcohol intake, which closely resembles that of human consumption [48]. When it comes to examining the effects of excessive alcohol exposure on behavior and the brain, humans differ substantially from rodents neuroanatomically and physiologically, and in terms of lifespan, developmental stages, and social behavior, such that not all aspects of the human condition can be adequately modeled in rodents. Non-human primates provide extremely valuable data in this regard (e.g. longer lifespans, parallel developmental stages, complex social and affective behavior)[49].

Alcohol self-administration studies in humans benefits from strong face validity. Two main types of oral self-administration paradigms are used in humans. In the first, participants sample several placebo or alcohol beverages over four separate sessions and subsequently choose between the sampled beverages [50, 51]. The primary outcome is the number of alcohol beverages chosen and the percent of participants who consumed alcohol. In the second and more prevalent paradigm, participants make a choice between money and alcohol [52]. After a priming dose, participants are presented with a tray of four alcohol drinks standardized to body weight/sex and are instructed that that they may consume as many drinks that they like over the next hour, or receive monetary compensation for drinks not consumed. The primary outcomes include the number of drinks consumed, observed BAC level, and percentage of subjects who abstained. While these procedures offer strong face validity in the physical consumption of alcohol; there are some limitations which may reduce the predictive validity of these tasks. Specifically, laboratory human alcohol self-administration paradigms are sometimes conducted in non-stimulating settings, where the choice to drink may present the only stimulating option. This is in contrast to both the rodent experience, where self-administration occurs outside the home cage and provides novel stimulation, and that of the human condition where drinking can occur in a number of stimulating environments. Human studies also often employ the use of a bar laboratory setting to increase the ecological validity of the self-administration. While no “bar-like” laboratories exist for rodent models, evidence indicates that rearing [53] and housing [54] conditions influence the reinforcing aspects of alcohol and consumption, demonstrating the importance of environment on self-administration. Despite limitations, pharmacological evidence provides support for the predictive validity of these paradigms. Medications known to reduce drinking in individuals with and AUD also reduce drinking in laboratory self-administration paradigms, indicating that these paradigms may be a useful tool for identifying therapeutic targets [35].

Intravenous alcohol self-administration is also possible using CAIS. The most widely used CAIS paradigm uses a free-access or fixed-ratio 1 schedule of reinforcement. To begin, priming doses are usually ordered over a 10-minute period followed by a 15-minute waiting period. Primary outcome measures are the number of drinks ordered and peak BAC. The CAIS can accommodate various schedules of reinforcement including progressive ratio schedules discussed below. Furthermore, a sustained attention task can also be incorporated in to CAIS, where rather than just pressing a button, participants must press and hold a response button and release within a short window of time in order to receive an alcohol infusion [55]. While control of BAC and alcohol cues offer distinct advantages, the CAIS implements a BAC safeguard that temporarily inactivates the response button at a pre-specified BAC level. The CAIS paradigm is limited by its low ecological validity, as well as substantial costs and effort to implement. Together, both animal and human self-administration models have strong potential for translation with the key recommendation being that experimental design be maximally informed by one another, resulting in a very similar set of parameters and responses.

#### Acquisition and maintenance

Acquisition of alcohol self-administration is defined as initial use of alcohol, a transition from early sporadic sampling to an increase in consumption over a period of hours, days, or weeks culminating in a steady rate of intake over time [56]. The use of animal models to represent this phase of problematic alcohol use in humans has many advantages. These studies may then provide information regarding the initiation pathological alcohol drinking in humans on factors regulating the initial occurrence of drug addiction, the underlying neurobiology, and how drug addiction can be prevented. As expected, a combination of biological and environmental factors influences acquisition of alcohol self-administration. Female rodents and non-human primates acquire alcohol self-administration faster than males [57, 58]. Additionally, exposure to alcohol in adolescence enhances acquisition of alcohol self-administration in adulthood [59]. It is also well known that adolescent rats acquire alcohol self-administration faster than adults [56] and that rats with greater sweet taste preferences acquire alcohol self-administration faster [60], suggesting baseline differences in reward sensitivity. Genetic breeding for high alcohol intake results in rapid acquisition of alcohol self-administration [61]. And like stress-induced drinking in humans, exposure to forms of social stress increases the acquisition of alcohol self-administration in rats [56]. This phase of operant alcohol self-administration lacks a direct human laboratory analog (see Table 1).

After lever pressing for alcohol has been established, pharmacological and schedule manipulations can be used to examine effects on maintenance responding. For example, under progressive ratio schedules, the response requirement to obtain an alcohol delivery gradually increases during the operant session (see Fig. 2). The maximum number of responses the animal makes in order to receive alcohol is referred to as the break point. This type of schedule reflects an animal’s motivation to obtain an alcohol delivery. Breakpoints are higher when assessed in alcohol withdrawal [62] and in female rats [63]. In addition, rodent and non-human primate studies incorporate these schedules to assess the impact of potential therapeutics on reducing motivation for alcohol [64–66].

Progressive ratio paradigms have also been used in translational human studies to closely mirror behavioral paradigms used in animal models (see Fig. 2). These studies have identified novel predictors of alcohol motivation. In non-treatment-seeking heavy drinkers, AUD severity and alcohol craving were strongly predictive of alcohol self-administration in an IV alcohol progressive ratio paradigm [67]. In a follow-up to this study, there was a clear separation between alcohol-motivated and alcohol unmotivated individuals over the course of the trial. Machine learning methods suggested that craving during alcohol administration and delay reward discounting were two robust predictors of motivation for alcohol [68]. Thus, the progressive ratio model combined with the IV alcohol administration shows promise as a translational paradigm (see Table 1).

Maintenance of alcohol-related behavior may be a complex phenotype in humans. Negative urgency, an impulsivity trait defined as risk taking during negative emotional states, is predictive of motivation for alcohol (i.e. breakpoint) during negative mood induction [69]. Sex differences in motivation to work for alcohol have also been reported [70, 71], which may be impacted by duration of abstinence prior to the study [70]. In an alternate approach, motivation for alcohol has been characterized by classifying based on their number of self-infusions, measured via button press, during the initial phase of a free-access IV alcohol paradigm [72]. High responders reported heavier drinking patterns, had lower negative alcohol expectancies, and were more impulsive than low responders [72]. Alcohol self-administration under progressive ratio conditions can be even more informative than self-administration models in that a standardized and translational schedule of reinforcement may facilitate cross-species analyses of behavior. The fact that experimental pharmacology in humans permit self-administration under a progressive ratio schedule speaks to efforts on both sides of the translational spectrum to ‘sync’ paradigms and behavioral outcomes for the benefit of clinical research.

### Experimental paradigms used to model alcohol craving and relapse

Part of what contributes to the chronic and relapsing nature of AUD is that alcohol-associated stimuli can increase alcohol craving, and as a result, trigger relapse to alcohol drinking in abstinent individuals with AUD [73, 74]. Conditioning models have been developed to reflect this phenomenon in animals.

#### Reinstatement

Conditioned reinstatement refers to the resumption of extinguished operant responding induced by noncontingent exposure to an alcohol-related cue [75, 76]. The most widely employed animal model of craving and relapse is the extinction/reinstatement model, wherein operant responding for alcohol is extinguished prior to reinstatement sessions [77]. The validity of this approach has been challenged, and the literature reflects both supportive and critical appraisals of the procedure. With appreciation of the limitations and advantages of this paradigm, reinstatement procedures are critical for investigating the neural basis of craving and relapse and for evaluating the potential of pharmacological treatments for the prevention of alcohol craving and subsequent relapse analog in rodents. These models are highly relevant as craving remains one of the most biologically-based symptoms of AUD according to DSM-5.

#### Cue-induced reinstatement (discrete and discriminative)

**Fig. 3:**
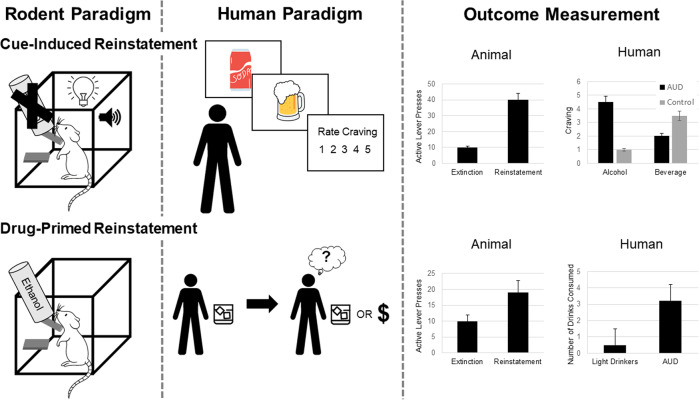
Depicts rodent and human paradigms and outcome measurement for cue-induced and drug-primed reinstatement. In rodents, exposure to alcohol-associated contextual stimuli (or an alcohol-paired environmental context) reliably elicits recovery of extinguished responding at a previously alcohol-paired lever without further alcohol availability. The conditioned effects of these stimuli are resistant to extinction in that recovery of alcohol-seeking does not diminish when these cues are presented repeatedly under non-reinforced conditions. In humans, presentation of visual or olfactory alcohol cues are used to induce craving. For drug-primed reinstatement, rodents who have previously undergone extinction training are exposed to a small dose of alcohol to measure the reinstatement of alcohol seeking. In humans, a small dose, or “priming” dose of alcohol is consumed to elicit craving and model “loss of control” over drinking.

Both response-contingent and response-noncontingent exposure to alcohol-associated contextual stimuli (or an alcohol-paired environmental context) reliably elicits recovery of extinguished responding at a previously alcohol-paired lever without further alcohol availability (see Fig. 3) [78, 79]. The conditioned effects of these stimuli are resistant to extinction in that recovery of alcohol-seeking does not diminish when these cues are presented repeatedly under non-reinforced conditions [80], and can even increase in magnitude over time, a phenomenon that has been referred to as “incubation of craving” [81, 82]. Consistent with clinical findings, reinstatement induced by alcohol cues is sensitive to reversal by opioid antagonist administration [83, 84]. For example, in individuals with AUD, naltrexone attenuates cue-induced craving [85] and reduces relapse rates [86, 87]. Moreover, correspondence exists between neural mapping data in animals [88, 89] and functional brain imaging studies in drinkers (e.g., [90, 91] with respect to the neurocircuitry activated by alcohol cue manipulations that, in humans, is closely linked with self-reports of craving. Alterations in the neurocircuitry responsible for cue-induced craving are associated with an increased susceptibility to relapse [92]. Conditioned reinstatement of ethanol-seeking in animals, therefore, has good ecological validity as a model of craving and relapse linked to alcohol cue exposure. One of the criticisms of cue-reactivity models in humans is the notion that not all individuals are cue-reactive [93]. A similar process may be in place with the animal phenotype of sign-trackers versus goal trackers, whereby only a subset of animals is deemed a sign-tracker. While controversies exist, the notion of cue-induced reinstatement and relapse provides one of the more robust models of AUD with important treatment implications. The consilience between animal and human neurocircuits subserving craving suggests that better translational models of AUD phenomenology may result in more consistent findings across species (see Table 1).

#### Alcohol-primed reinstatement

Small doses of alcohol can elicit rather than suppress alcohol craving. In individuals with AUD, the first drink after a period of abstinence has long been associated with “loss of control” leading to intoxication and return to pathological alcohol drinking [94]. This priming effect can be recapitulated in animals(see Fig. 3) [95]. However, the effect of alcohol priming on reinstatement is modest, dose-dependent, and relies on the concurrent presentation of alcohol-associated stimuli [96]; therefore, the use of this procedure to model the “loss of control” phenomenon seen in humans is questionable. Several human laboratory models use a priming period prior to self-administration or decision making in order to provoke drug-primed reinstatement model in humans. It has been documented in the clinical literature that individuals tend to have a binge episode when they consume any alcohol and violate an expectation of abstinence (i.e., Abstinence Violation Effect, AVE). This is another area in which translation and reverse translation could be enhanced, particularly in the context of treatments that do not have abstinence as a primary endpoint. The acceptance of endpoints related to controlled drinking and psychosocial improvements is gaining traction in clinical samples [97] and could lead to a host of preclinical experiments. For instance, the use of lickometers (also called drinkometer), which are more sensitive to consumption patterns by capturing the microstructure of licking, not just total volume [98], may be a useful addition to preclinical experiments. This may be especially useful in conjunction with in vivo brain measures (e.g., electrophysiological or calcium imaging data) and to measure more subtle changes in response to brain manipulations (e.g., optogenetics, chemogenetics).

#### Stress-induced reinstatement

**Fig. 4:**
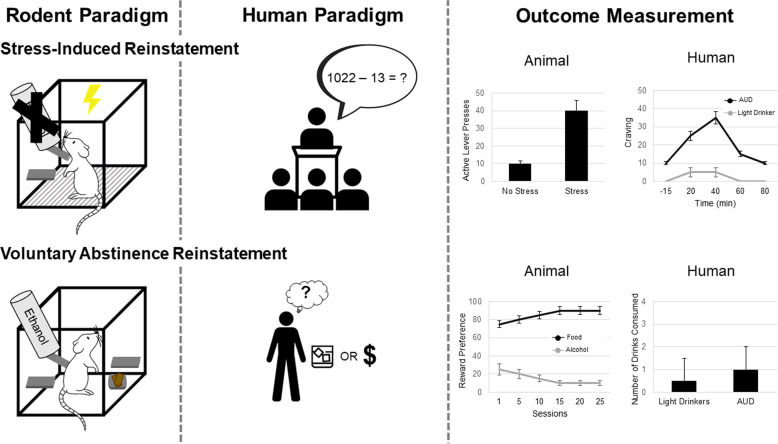
Depicts rodent and human paradigms and outcome measurement for stress-induced voluntary abstinence reinstatement. The most predominate model of stress induction in rodents is footshock, where a rodent who previously extinguished responding for alcohol returns to alcohol seeking after exposure to footshock stress. In humans, one common paradigm to induce alcohol craving is the Trier Social Stress Task, during which participants must deliver a speech and perform mental arithmetic in front of an unsupportive audience. To model voluntary abstinence in rodents, animals that have been trained to self-administer both food and drugs are given daily mutually exclusive choice trials between a drug and a palatable food. Preference for food or alcohol is used to measure the choice for alcohol over alternate rewards. In humans, voluntary abstinence can occur in the presence of alternative rewards, such as a maintaining a job, relationships, and health.

Stress is a major driver of AUD symptomology in humans and a determinant of relapse [99]. The role of stress in alcohol-seeking is well-established in the animal literature. Physical, social, and emotional stress can facilitate acquisition or increase alcohol self-administration in rodents and nonhuman primates [77, 100]. Stress elicits reinstatement of alcohol seeking in alcohol-free animals, with footshock being the most predominate method of stress-induction (see Fig. 4) [81]. In the original set of studies [101, 102], rats were trained to self-administer alcohol and reinforced responses were paired with a noncompound (simple) conditioned stimulus. After a withdrawal period, alcohol-reinforced responding was extinguished, and reinstatement of alcohol-seeking was investigated under three conditions: (1) during response contingent presentation of the CS alone; (2) after exposure to a 10 min footshock stress period; (3) during response contingent presentation of the CS following exposure to footshock stress. Alcohol CS and foot shock alone resulted in threshold effects on alcohol-seeking behavior. However, the alcohol CS elicited strong responding in animals that had been subjected to footshock stress before the session. Thus, interactive effects between alcohol cues and stress-exacerbating alcohol seeking can readily be demonstrated in animal models.

Non-human primate models can be used to bridge the translational gap between rodent and human studies investigating the interaction between stress and excessive drinking. Social rank and social setting can be used to induce stress in non-human primates; where animals undergoing social isolation stress consume greater amounts of alcohol than peer-reared monkeys [103]. Non-human primates can also be used to evaluate the effect of early-life stress on the development of future alcohol consumption. Moreover, these longitudinal non-human primate studies can address the importance of the developmental stage during exposure to stress, the nature of early life stress, and/or the chronicity of the early-life stressor in the risk for developing excessive alcohol consumption [103].

Significant progress has been made in the translation of this work to human samples. Two paradigms have been applied to examine the role of stress on craving and relapse in humans: guided imagery stress and the Trier Social Stress Task. In the guided imagery stress paradigm, individualized stress, drug-craving and relaxation scripts are developed and then presented to an individual in a counter-balanced order [104]. This paradigm has reliably induced craving in individuals with substance and alcohol use disorders [92, 100, 105, 106]. Critically, stress-induced craving is predictive of time to alcohol relapse, number of drinking days, and number of drinks consumed after alcohol treatment [107–109]. Moreover, biobehavioral stress response in the guided imagery task, measured through hypothalamic-pituitary-adrenal (HPA) axis response, is also predictive of alcohol relapse, such that a lack of stress-induced corticotropin and cortisol responses predicted a shorter time to alcohol relapse [109]. The Trier Social Stress Test (TSST) is designed to induce stress in the laboratory through a combination of stressors in which participants must deliver a speech and perform mental arithmetic in front of an unsupportive audience (see Fig. 4) [110]. In healthy individuals, the TSST induces changes biological measures of stress, including increasing cortisol levels and heart rate [110]. The TSST has been used to evaluate the effect of stress on a variety of behaviors in individuals with AUD, including motivation for alcohol [111], the incentive value of alcohol [112], and in-lab oral alcohol consumption [113]. In individuals with AUD stress-induction through the TSST increases alcohol craving [111, 112, 114], but see [115]. Furthermore, individuals with AUD who later relapse show marked reductions in stress-induced cortisol response during the TSST compared to abstainers and healthy controls [116]. Several human laboratory and randomized clinical trials have been conducted to examine pharmacotherapies targeted at reducing stress and alcohol craving (reviewed in Mantsch, Baker, Funk, Lê and Shaham [117]), which are beyond the scope of this review, yet exemplify the translational value of the stress-induced reinstatement animal model. Similar to cue-induced craving, it is possible that only a subset of individuals is sensitive to the stress-induced effects on craving and/or relapse. In fact, studies have found that medications working on stress-systems may be most beneficial to those who are most stress-reactive [118].

#### Abstinence-based models

Traditional reinstatement models have the limitation that the abstinence period in humans is not due to operant extinction but is either forced or voluntary due to the presence of alternative rewards and/or negative alcohol-related consequences. Thus, animal paradigms were developed to include facets that mimic abstinence in humans that do not include extinction training. One of the opportunities for reverse translation may be models in which instead of abstinence, the animal is exposed to moderate amounts of alcohol, thus mimicking “controlled drinking.” Little has been done in that domain from the preclinical perspective. Given the well documented pattern of gradual relapse in humans, there is tremendous potential to reverse translate a controlled drinking paradigm in rodents. This may be helpful in understanding mechanisms of action of pharmacotherapies, since some medications may be more effective at reducing heavy drinking while others can promote abstinence as a primary outcome.

#### Forced abstinence and incubation of craving

Animal models of relapse have been conducted after forced abstinence in which alcohol seeking progressively increases after the cessation of alcohol administration, a phenomenon termed “incubation of alcohol craving”. In this model, rats are first trained to self-administer alcohol and are then tested for nonreinforced alcohol seeking at different time periods of forced abstinence. During relapse tests, rats are brought back to the self-administration environment and lever presses lead to contingent presentation of discrete cues paired with alcohol administration [119]. Clinical studies show that the incubation phenomenon is similarly present in humans with substance and alcohol use disorder exposed to drug-paired cues during abstinence [120–122]. When paired with neuroimaging methods, the incubation of craving phenomenon in humans can be neuroscience-informed and yet clinically useful. Specifically, neuroimaging can be used to measure neural incubation of craving using a functional magnetic resonance imaging alcohol cue reactivity paradigm. In patients undergoing early abstinence, cue reactivity increased in the striatum [123]. Patients treated with naltrexone had attenuated cue-reactivity, i.e., did not show an incubation of craving, and had a reduced risk for relapse during the first 3 months of treatment [123]. This study demonstrates the potential role of neuroimaging in understanding psychological constructs, including incubation of craving and may promote neuroscience-informed reverse-translation efforts. As discussed above, the chronic nature of AUD may be, at least in part, due to memory-related alterations that render individuals vulnerable to relapse even after long periods of abstinence.

#### Voluntary abstinence by introducing adverse consequences

The limitation of the rat incubation of drug-seeking model is that the abstinence period is experimenter imposed or forced. Abstinence in humans is often voluntary and may be due to negative alcohol-related consequences [124, 125]. Research in this area has primarily involved punishment (shock delivered after operant response)- and conflict (electric barrier is in front of drug-paired lever))-based relapse models. In both models, operant self-administration is suppressed by an aversive shock before the relapse tests. Thus, animals are presented with a conflict between desire for the drug and its adverse consequences. Alcohol studies using punishment-induced abstinence procedures have primarily been conducted in rats [126, 127].

#### Voluntary abstinence by introducing non-drug rewards in discrete choice procedures

Abstinence in humans can also be voluntary due to the presence of alternative rewards [124, 125]. In this procedure, animals that have been trained to self-administer both food and drugs are given daily mutually exclusive choice trials between a drug and a palatable food [128]. Incubation of drug craving occurs after the alternative drug reward is discontinued. The magnitude of the incubation test is a function of time, as higher methamphetamine-seeking behaviors are seen after 21 days compared to 1 day of voluntary abstinence [129]. This procedure has been combined with the punishment procedure (reviewed above) to investigate incubation of craving in rats [130]; sex differences were reported, such that resurgence of alcohol seeking occurred in both sexes following the removal of the combined alternate reinforcer and punishment, but only occurred in female rats when the punishment alone was removed.

### Experimental paradigms to model inflexible stimulus-reward learning and value-based decisions

#### Reversal learning

**Fig. 5:**
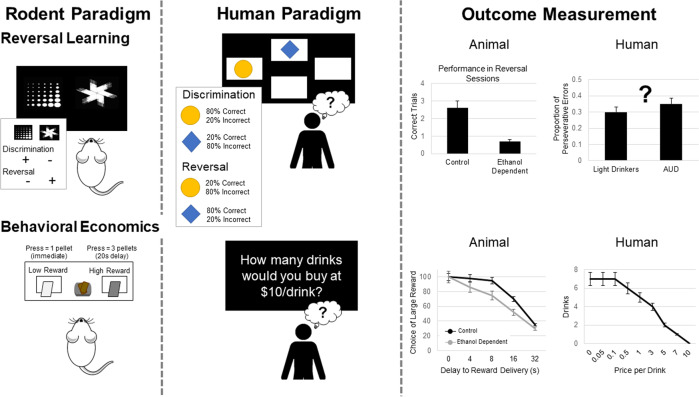
Depicts rodent and human paradigms and outcome measurement for reversal learning and behavioral economics. In rodents, stimulus-based deterministic (fully-predictive) reversal learning paradigms are often used. In these paradigms, rodents first learn to discriminate and choose between two stimuli, one of which is rewarded and the other which is not. After successful discrimination, the associated outcomes between the two stimuli are reversed, such that the previously rewarded stimulus now becomes the non-rewarded stimulus and vice versa. In humans and non-human primates, probabilistic reversal learning paradigms are often used, in which the two stimuli are associated with a probability of reward (e.g., 0.80 vs. 0.20). The stimulus associated with a greater probability of reward is considered the “better” option, and the stimuli with the lower probability is considered the “worse” options. Reversal learning paradigms are used to measure cognitive flexibility, which is often impacted in AUD. The use of reversal learning paradigms has been limited in the human model and additional translation is needed. Rodent behavioral economics paradigms involve the choice of a larger/later reward over a smaller/sooner reward. In these paradigms rodents learn to level press for a palatable food reward; one lever is associated with a smaller reward delivered immediately, while the other lever is associated with a larger reward delivered after a delay. In humans, behavioral economic paradigms typically ask a series of questions regarding a participant’s willingness to purchase alcoholic drinks at a series of costs. Demand curves can be generated for alcohol rewards that dissociate the value at the lowest effort cost from a subject’s sensitivity to increasing cost.

Individuals with AUD show cognitive impairments, including in the domain of cognitive flexibility, broadly defined as the ability to adjust one’s behavior in response to changes in the environment [131]. In humans, cognitive flexibility is commonly assessed using the Wisconsin Card Sorting Task [132]. Individuals with an AUD show impairments in cognitive flexibility by committing more perseveration errors after the classification rule has changed [133, 134], which may reflect their inability to disengage from patterns of problematic drinking and adopt more effective behavioral strategies. Reversal learning is another robust measure of flexibility with excellent cross-species convergence in terms of neural substrates [135], with many different experimental variants, and is frequently probed experimentally in animals. In stimulus-based deterministic (fully-predictive) reversal learning paradigms, subjects first learn to discriminate and choose between two stimuli, one of which is rewarded and the other which is not (see Fig. 5). After successful discrimination, the associated outcomes between the two stimuli are reversed, such that the previously rewarded stimulus now becomes the non-rewarded stimulus and vice versa [135–137]. In probabilistic reversal learning paradigms, the two stimuli are associated with a probability of reward (e.g. .80 vs. .20, .70 vs. .30), the stimuli associated with a greater probability of reward is considered the “better” option, and the stimuli with the lower probability is considered the “worse” options [135, 137–139]. Reversal learning paradigms requiring subjects to remap reward contingencies are often used in animals to test the effects of alcohol on flexibility [137, 140–143]. Investigators have tested the effects of forced alcohol exposure on reversal learning using intragastric gavage [140–142], intraperitoneal injections [140], and alcohol by passive vapor inhalation [43, 134, 144, 145]. We recently reported that chronic intermittent voluntary alcohol consumption in rats has the most impact on attentional measures in subsequent early learning and exploration strategies (Win-Stay, Lose-Shift) during reversal learning, rather than an effect on omnibus, motivational measures of reversal learning per se [137]. Cognitive flexibility has also been measured in non-human primates to evaluate risk for future heavy drinking. Low cognitive flexibility assessed through a set-shifting task predicted future heavy drinking during late adolescence in rhesus macaque monkeys [146]. Thus, results have been mixed and largely dependent on method of administration and animal model, such that it remains unclear whether alcohol experience impairs learning flexibility (i.e., reversal learning, specifically) in animals.

Interestingly, support for reversal learning and cognitive flexibility impairments in human models of AUD is modest at best. In order for these models to be maximally useful, it is critical to establish their explanatory value vis-a-vis addiction phenomenology constructs. In other words, the cognitive neuroscience application of these models has yet to be expanded to a clinical neuroscience direction in which these cognitive phenotypes are more directly mapped to clinical phenomena in AUD. To date, much of the relevance is inferred rather than demonstrated through experimental studies (see Table 1).

#### Behavioral economics and alternative rewards

Chronic exposure to alcohol or other drugs may induce neuroplastic changes that contribute to an enduring phenotype of aberrant evaluation of reward costs, or cost sensitivity. There are several types of value-based decision-making paradigms that have been probed in rodents: delay-, effort-, and risk- costs, to name a few [147]. Generally, these behavioral tests involve the choice of a larger/later reward over a smaller/sooner reward, or a preferred, but costly, high-value reward vs. a less-preferred, low-cost, low-value reward (see Fig. 5). A common practice in analysis of experimental animal data is fitting behavioral choices to either exponential or hyperbolic functions (specifically *k* parameter, a measure of how steeply the cost decreases the value of a reward), measuring indifference points (i.e., the point at which the two options are subjectively equivalent), or break points (i.e., the point at which the animal ceases to pursue the costly option). Such decisions involving relative value comparisons, or economic or value-based decisions, do not probe learning as above but rather they instead test performance based on established preferences in animals.

Behavioral economic indices are often used to assess drug-taking and seeking in humans and nonhumans [148–150], but can also be used to estimate how value systems can change following chronic drug and alcohol experience. For example, demand curves can be generated for either natural (food) or drug rewards that dissociate the value at the lowest effort cost (Q_0_) from a subject’s sensitivity to increasing cost (α), as well as other measures of demand inelasticity derived from α, or essential value (EV; see Fig. 5) [151]. Chronic psychostimulant experience, either experimenter-administered [152] or self-administered [149], results in enhanced cost sensitivity even though hedonic value (i.e. value at no cost) remains intact. This suggests that drug experience can recalibrate reward circuits to render behavior biased in favor of easier and shorter-term goals [153]. This has implications for the value of long-term sobriety and abstinence. To our knowledge, a behavioral phenotype has not been uncovered following chronic alcohol experience. Nevertheless, the behavioral economic literature has grown considerably in the field of AUD, and addiction broadly, over the past two decades. Demand curves have been used to map behavioral responses in humans and these approaches provide unique opportunities for translation. Lacking is robust demonstration that behavioral economic indices in fact predict clinical outcomes, including treatment response (see Table 1).

## Conclusions and future directions

Most of the animal models discussed above have face validity to some distinct aspect of AUD. Perhaps more important that face validity, these models capture a phenomenon that is akin to the addiction phenomenology, an inherently human process. In addition, the measures are stable and consistent, with little within-subject and between-subject variability. Of course, the fact that animal models cannot parallel the complexity of human life cannot be understated. Factors such as drug availability, economic, and socio-cultural levels influence the opportunities and experiences available to individuals and thus impact the risk of AUD. Preclinical models that implement alternative rewards and behavioral economics begin to address these factors, but these models are not widely used in alcohol research. A common criticism of animal models is that they have not contributed to novel treatments for AUD. The last medication for AUD with a novel mechanism of action was acamprosate, which was FDA approved in 2004. While improvements in the generalizability and rigor of animal models are warranted, the utility of animal models in identifying novel therapeutics for neuropsychiatric conditions should not be discounted. For instance, basic research was instrumental in identifying ketamine’s mechanism of action as a novel medication for major depressive disorder. The lack of effective medications for AUD does not rest with animal models alone, but all approaches in psychiatry. Compared to other organ systems, the human brain and neuropsychiatric disorders are unique and complex, which makes identification of novel therapeutics difficult to establish.

Animal models have identified many biological targets that could potentially be targeted for intervention. We contend that there needs to be more stringent criteria and more robust evidence to validate these drug targets prior to advancing them towards translation to humans. Deep phenotyping of individuals with AUD may offer important insights into understanding its heterogeneity. Currently, AUD is diagnosed using clinical criteria that does not underly specific brain mechanisms. At times, the clinical criteria for AUD are not objective; thus, outcomes would benefit from more laboratory-based studies of human subjects to identify quantitative phenotypes that are treatment responsive. Identification of quantitative phenotypes in humans will encourage reverse translation in animals [154]. Collectively, the field should better integrate these various levels of analyses with a sharper focus on advancing basic discoveries into the clinic. A clinical neuroscience framework argues for the validation of basic neuroscience constructs through their integration with clinical phenomenology, including etiological and treatment models of addiction [155].

To improve the predictive validity of preclinical work in medications development, we suggest that animal models of AUD focus on behavioral endpoints that have direct translational value to AUD in humans. The same is true for human clinical research where alcohol intake endpoints should be considered the gold standard in clinical assessment. It is rational and advantageous for preclinical work to focus on behaviors that parallel key aspects of AUD phenomenology in order to improve their generalizability. Equally important, animal models should not only reflect behavioral features of human AUD, but also capture the neurobiological dysfunctions, which makes AUD a chronic, relapsing disorder. Given that only a fraction of individuals who drink will develop an AUD, only a fraction of rats trained to lever press for alcohol will display enhanced motivation and compulsive alcohol-seeking behaviors [156]. Focusing research efforts on this subset of vulnerable animals may help elucidate the heterogeneity of AUD. Lastly, we echo calls for changes in animal models that can be employed immediately to improve preclinical-to-clinical translation. Experimental design decisions (i.e., appropriate statistical power, treatment groups matched for litter and sex, treatment blinding) should be seriously considered to improve replicability in preclinical studies. The regular reporting of descriptive statistics and effect sizes will also aid in increasing the rigor and reliability of preclinical data. This approach can also inform big data analyses at the level of animal experiments.

Advancements in the clinical side of the translational spectrum are also in order. Notably, a popular model of AUD argues for a transition from reward-driven to stress-driven drinking [26, 157–159]. Human studies to date have largely been unable capture the “dark side” of addiction [67, 160–162], marked by excessive drinking to alleviate stress and withdrawal. This has led clinical scientists to rethink the sample selection, including the lack of treatment-seekers for AUD in experimental medicine studies [163, 164], which may give rise to samples that are not fully representative of the addiction spectrum, and the dark side, in particular. Such translation challenges may also account for the null findings in the development of the promising CRF antagonist compound [165], thought to target stress-dysregulation in later stages of addiction. The lack of representation of more severe AUD phenomenology in clinical samples may also result in our inability to capture the withdrawal state and therapeutic effects on protracted withdrawal, as clinical studies most often exclude individuals experiencing clinically-significant withdrawal. Clinically, the distinction between the management of alcohol withdrawal during the detoxification phase of treatment versus the maintenance phases limit the translation of compounds that can target protracted withdrawal. Sex-dependent effects represent another missed opportunity in translational science of AUD. Both preclinical and clinical studies often lack the representation of female participants/subjects and robust conclusions about sex-effects cannot be drawn with such inconsistent literature and mixed methods. In brief, both clinical and preclinical models must achieve sufficient phenomenological and methodological overlap to effectively test hypotheses across levels of analyses. This includes accurate representation of female participants as well as representation of the target phenomenology in the study sample, such as alcohol withdrawal, clinical severity spanning the full spectrum of AUD, and stress-system dysregulation in late-stage AUD.

In closing, the use of laboratory animals to study human disease states, including AUD, continues to have significant merit. It remains inconceivable that new treatments could be developed and successfully advanced to testing in humans without some degree of validation in laboratory animals. It is our hope that the dissection of animal and human analog models provides some clarity as to why these models may/may not be well suited for translation. A focus on the ‘big picture’ requires prioritizing animal and human models that are most proximal to core features of AUD. Lastly, the recommendations provided herein will increase healthy discussion and provide a starting point towards refining interpretation of animal models, which themselves do not represent AUD, but are *useful* in the study of AUD and its treatment in humans.
